# Weaned Sows with Small Ovarian Follicles Respond Poorly to the GnRH Agonist Buserelin

**DOI:** 10.3390/ani10111979

**Published:** 2020-10-28

**Authors:** Tania P. Lopes, Lorena Padilla, Alfonso Bolarin, Heriberto Rodriguez-Martinez, Jordi Roca

**Affiliations:** 1 Department of Medicine and Animal Surgery, Veterinary Science, University of Murcia, 30100 Murcia, Spain; taniamarisa.piedade@um.es (T.P.L.); lorenaconcepcion.padilla@um.es (L.P.); 2 AIM Iberica, Topigs Norsvin España, 20290 Madrid, Spain; abolarin@aimiberica.com; 3 Department of Biomedical and Clinical Sciences (BKV), Linköping University, SE-58185 Linköping, Sweden; heriberto.rodriguez-martinez@liu.se

**Keywords:** weaned sows, ovarian follicles, ovulation synchronization, buserelin

## Abstract

**Simple Summary:**

This study evaluated the influence of mean ovarian follicle size and the season of weaning on the effectiveness of administering the GnRH agonist buserelin to synchronize ovulation in weaned sows. The results from 352 sows demonstrated that sows with small follicles (<0.5 cm in diameter) at treatment are poor responders, a condition more frequent among sows weaned in summer–autumn than in those weaned in winter–spring.

**Abstract:**

The GnRH agonist buserelin (GnRH), used to synchronize ovulation in weaned sows, attains only 70–80% effectivity, owing to several reasons of ovarian origin. This study evaluated in particular whether mean ovarian follicle size at treatment and the season of weaning are among those influencing GnRH responsiveness. The experiment was carried out in a temperate-region farm with 352 sows of 1–6 parities weaned either in winter–spring (WS, 174 sows) or in summer–autumn (SA, 178 sows). The sows were randomized into two groups: GnRH (10 µg of buserelin acetate at 86 h after weaning, 172 sows) and control (180 sows). The ovaries were transrectally scanned from weaning to ovulation and the sows clustered according to their mean follicular size at treatment time: small (<0.5 cm in diameter), medium (0.5 to 0.64 cm) and large (0.65 to 1.09 cm). In total, 88.33% of the GnRH-treated sows ovulated, with 82% of them within the expected time window (120–132 h after weaning). In contrast, 95.45% of the unresponsive sows had small follicles at the time of treatment and were mostly weaned in SA (20.45%) than in WS (4.76%). In conclusion, the conspicuous presence of sows having small ovarian follicles at treatment time compromises the efficiency of the GnRH agonist buserelin to synchronize ovulation in weaned sows, which occurs more frequently in summer–autumn weaning.

## 1. Introduction

Artificial insemination (AI) is widely used in the swine industry, becoming an essential tool to improve production [1,2], yet still requiring improvements to be fully efficient [3]. In this context, the implementation of a single fixed-time AI without the need of detecting estrus instead of customary multiple AIs after estrus detection offers substantial benefits. For instance, it eases management [4,5,6,7], saves labor and money and generates fewer environmental pollutants [1,5,8], alongside easing the optimal use of boars with a high genetic index. Yet, in order to achieve the highest fertility outcomes, effective single fixed-time AI requires inseminating just before the expected ovulation time. This obvious prerequisite is not easy to fully achieve in commercial settings since the timing of spontaneous ovulation is highly variable among sows, especially among weaned sows [7,9]. Consequently, controlling ovulation time is essential for scheduling the best timing for a fully effective single fixed-time AI.

Currently, exogenous hormones have proven practical utility for successful induction and synchronization of ovulation time in sows, inducing ovulation within an expected narrow time window in most sows [8,10]. The useful hormones include human chorionic gonadotropin (hCG; [11,12,13]), porcine luteinizing hormone (pLH; [14,15]) and GnRH analogues, particularly synthetic agonists such as buserelin [16,17,18], licerelin [19] or triptorelin [20,21]. GnRH analogues are currently the most used since they induce an endogenous LH surge that is quasi-physiological, they can be administered by different routes and several synthetic agonists are commercially available with different bioactivities and half-lives [22]. However, they do not reach high and consistent synchronization results in weaned sows, as the percentage of sows ovulating within the expected time window are usually below 80% and vary substantially among studies, ranging from 57.9% [20] to 76% [17]. The timing and route of administration, together with the number of previous parities, are among the so-far proven causes explaining the faulty and variable synchronization response [17,20]. However, other causes have been poorly evaluated, such as the size of the ovarian follicles at the time of treatment and the season of weaning, which could also influence GnRH responsiveness.

To our knowledge, only Knox et al. [20] evaluated the influence of the average ovarian follicular size at treatment time on the ovulation synchronization capacity of a GnRH agonist. Knox et al. [20] grouped the weaned sows into two clusters of either large and “not-large” follicles, a clustering far from the general consensus of separately weaned sows having small, medium, or large follicles [22]. Since weaned sows with small follicles have the worst reproductive outcomes, including delayed post-weaning ovulation [23], additional studies evaluating the influence of ovarian follicle size on the reproductive performance of weaned sows treated with GnRH agonists are still timely.

An additional factor to be considered, particularly in temperate regions, is that the reproductive performance of weaned sows is still influenced by the season of weaning; with sows weaned during summer and early autumn showing the worst reproductive performance, including delayed weaning-to-ovulation interval (WOI), than those weaned during winter and spring [24,25]. Whether or not a GnRH treatment modifies this seasonal reproductive pattern is unproven. As far as we know, only one study, yet carried out in the tropics with sows housed under artificially controlled environmental conditions, evaluated the influence of the season of weaning on the reproductive performance of GnRH-treated sows [18]. The environmental conditions defining seasons of year in the tropical region in question are very different from those of temperate regions, where pig production is more widespread. Furthermore, sows kept under artificially controlled environmental conditions may not fully experience the effects of potentially stressful external environmental conditions. Therefore, we need to include the influence of weaning season when studying the ability of GnRH treatment to synchronize ovulation in weaned sows in temperate regions.

With this background in mind, we hypothesize that sows with small follicles can be less sensitive to the GnRH agonist buserelin. Consequently, the aim of this study was to evaluate how the size of ovarian follicles at the time of treatment influences the efficiency of the GnRH agonist buserelin to induce and synchronize ovulation in weaned sows. To prove if the weaning season influences the response to buserelin, the sows under study will be weaned at two different periods of the year, namely autumn–winter and spring–summer.

## 2. Materials and Methods

### 2.1. Farm, Animals and Handling

The experiment was conducted in a commercial breeding farm with 2500 Landrace-Large White crossbred sows located in Murcia, southeast of Spain (37″59′ NL, 1°08 WL), with daylight varying from 14 h 48 min on the summer solstice to 9 h 32 min on the winter solstice. The maximum mean air temperature ranged between 32.8 (summer) and 17.7 °C (winter) during the year of the experiment.

The farm fulfilled the guidelines of the European Union in terms of production, health, biosecurity and animal welfare. The farm only had climate control in the farrowing rooms, where evaporative cooling systems and exhaust fans kept the ambient temperature around 24 °C. All the other farm facilities were open to environment temperature and natural light. At weaning, sows were placed in individual crates for oestrus detection, insemination and pregnancy diagnosis. Once pregnancy was confirmed by transabdominal ultrasound (28 days after the first insemination), and the pregnant sows were transferred to gestation open pens of 25 m^2^ (ten sows per pen) where they remained for up to 3–5 days before the expected day of farrowing. The sows were then placed in individual farrowing crates in climate-controlled farrowing rooms until weaning (20 sows per room).

The sows always had free access to water and they were fed with commercial feed that varied in composition according to the physiological state of the sow. While the composition for pregnant sows was 13% crude protein, 6.59% crude fat and 12.15 MJ ME/kg with a daily intake that varied from 2.3 to 2.8 kg, in lactating sows the composition was 17.50% crude protein; 4.17% crude fat and 13.4 MJ ME/kg with an average daily intake that increased progressively from farrowing to weaning (4.5 kg on average).

### 2.2. Estrus Detection and Insemination

Estrus was checked by trained farm staff always in the presence of a healthy mature boar placed in the alley in front of the sow crates, twice a day, starting on the second day after weaning. Sows were considered in estrus when they exhibited standing reflex to back-pressure test. Sows in estrus were intrauterine inseminated twice (at 0 and 24 h after the start of estrus), or three times (at 48 h) if they remained still in estrus, using the SafeBlue Foamtip^®^ with a PC Cannula device (Minitube, Tiefenbach, Germany). The liquid semen AI-doses used had 1.5 × 10^6^ total spermatozoa in 40 mL volume and they were provided by AIM Iberica (Topigs Norsvin España, Madrid, Spain).

### 2.3. Transrectal Ovarian Ultrasonography

Ovaries were scanned using a proven transrectal ultrasound procedure [26,27]. An ultrasound machine equipped with a 4–10 MHz multivariable frequency linear transducer (LOGIQ Book XP General Electric Co., Solingen, Germany) was used and all ultrasound scans were performed by the same researcher (T. Lopes). The ovaries were scanned following the procedure described by Bolarin et al. [28]. Briefly, the transducer was manually inserted into the rectum to a depth of 35–45 cm, to reach the expected anatomical ovarian location. The ovaries were separately scanned, recording a minimum of three video sequences per ovary, using the digital cinema technology provided by the ultrasound machine. Once in the laboratory, the videotapes were scrutinized by using frame-by-frame video playback to identify any physiological or pathological functional echoic structure present, which were counted and measured using the calibrated measurement software provided by the ultrasound machine. All thin-walled spherical anechoic structures up to 1.19 cm in diameter were considered follicles. The same structures, but with a diameter greater than 1.19 cm, were considered cysts. Hypoechoic circular structures were considered to be corpora lutea. Focusing on the follicles, the diameter (in cm) of the three largest follicles in each ovary was noted and the mean diameter of the 6 follicles in each sow recorded as the follicle size.

### 2.4. Experimental Design

**Figure 1:**
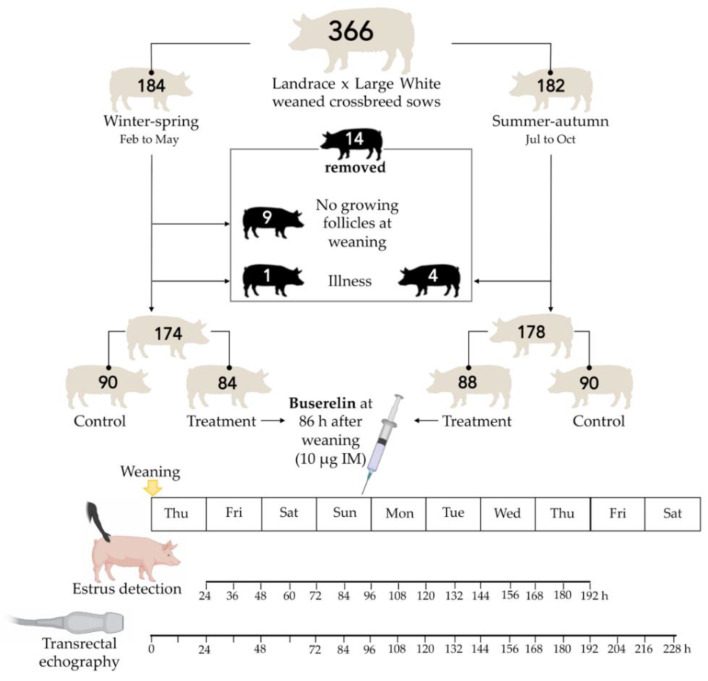
Flow chart showing the distribution of the sows between the periods of the year and the experimental groups together with the schedule followed for the detection of estrus and for the performance of the transrectal ultrasound of the ovaries.

The experiment was approved by the Bioethics Committee of the University of Murcia (research code: 639/2012). Figure 1 shows how the experiment was arranged. The experiment was conducted over eight months, specifically from February to May (winter–spring (WS) period) and from July to October (summer–autumn (SA) period). A total of 366 sows were chosen at weaning. Specifically, between 40 and 50 sows in each of the 8 months that the experiment lasted. Thus, a total of 184 sows were chosen during the WS period and other 182 during the SA period. Fourteen sows (four in SA and ten in WS) were removed from the experiment. Nine of the ten sows removed during WS showed cystic follicles (five sows) or corpus luteum (four sows) in the first ovarian scans carried out on weaning day. The other five sows were removed throughout the experimental period due to musculoskeletal disorders. Consequently, the experiment was finally carried out with 352 weaned sows (174 chosen in WS and 178 in SA) that were randomly allocated to a GnRH treatment group (172 sows, 84 in WS and 88 in SA) or an untreated control group (180 sows, 90 in WS and 90 in SA). The characteristics of GnRH-treated and control sows are showed in Table 1.

Sows of the GnRH treatment group received at 86 h after weaning an intramuscular 2.5-mL doses of Porceptal^®^ (MSD Animal Health, Kenilworth, NJ, USA), corresponding to 10 µg of buserelin acetate. The timing, dose and route of administration were those recommended by the manufacturer (MSD Animal Health) as demonstrated as efficient to treat weaned sows [17]. Ovulation should occur within a time window of 32–44 h after treatment because the response of sows is similar to that of endogenous LH surge [17]. Therefore, the expected ovulation time window would be between 120 and 132 h after weaning. The sows within the control group received an intramuscular 2.5-mL dose of saline solution at the same time after weaning as the treatment group sows. All sows were subjected to the detection of estrus twice a day (at 07:00 a.m. and 06:00 p.m.) from the day after weaning until eight days after weaning. Ovaries were scanned once a day (at 08:00 a.m.) from weaning to the onset of estrus, and thereafter twice daily (at 08:00 a.m. and at 07:00 p.m.) until ovulation. Sows were grouped into three clusters according the ovarian follicular size at treatment following the clusters recommended by Knox [22], namely small (<0.5 cm), medium (0.5 to 0.64 cm) and large (0.65 to 1.19 cm). Ovulation time was considered the scan moment where less than 50% of the follicles were counted compared to the previous scan. Sows that did not show signs of estrus during the first eight post-weaning days were considered in anestrus. From these data, the intervals from weaning to oestrus, from oestrus to ovulation and from weaning to ovulation (WOI) were recorded. The farrowing rate and the total number of piglets born per litter were also recorded.

### 2.5. Statistical Analysis

Data were analyzed by using IBM SPSS 24.0 (IBM Spain, Madrid, Spain). Pearson’s Chi-square test was used for comparing the distribution of sows among the different generated groups as well as for evaluating differences in farrowing rates. An unpaired t-test and Mann–Whitney test were used for evaluating differences in count data (duration of intervals and litter sizes) choosing one or the other depending on whether or not the data were normally distributed. Differences at *p* < 0.05 level were considered significant.

## 3. Results

### 3.1. Reproductive Performance of GnRH Agonist-Treated Sows vs. Untreated Controls

Twenty-two sows treated with the GnRH agonist (12.79%) and 21 control sows (11.67%) did not show estrus during the eight days after weaning. The GnRH agonist treatment did not modify the incidence of sows in anestrus. Most sows in anestrus were of one and two parities (50% (11/22) of GnRH agonist-treated and 66.67% (14/21) of control sows). None of the sows in anestrus ovulated. The percentage of weaned sows that ovulated was similar for the treated (87.21%, 150/172) and control (88.33%, 159/180) sows.

Regarding the recorded intervals, the weaning-to-estrus interval was similar for GnRH agonist-treated (102.6 ± 1.63 h) and control (108.40 ± 1.89 h) sows, while the estrus-to-ovulation interval and WOI were shorter (*p* < 0.001) for GnRH agonist-treated sows (32.40 ± 0.99 h and 134.88 ± 17.40 h, respectively) than for controls (39.85 ± 1.09 h and 148.23 ± 24.42 h, respectively). Focusing on WOI, 71.51% (123/172) of the all GnRH agonist-treated sows ovulated in the expected time window (120–132 h after weaning), while only 26.11% (47/180) of the control sows did so (*p* < 0.001). Regarding sows in estrus, the percentages that ovulated in the expected time window were 82.00% (123/150) and 28.93% (46/159) for treated and control sows, respectively (*p* < 0.001).

**Figure 2:**
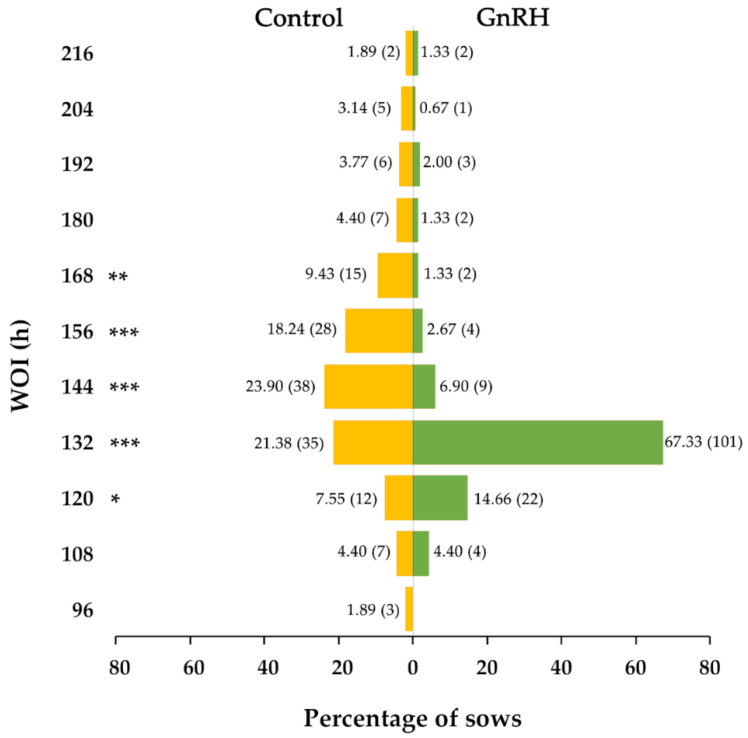
Distribution of the GnRH agonist-treated (GnRH) and the untreated control (Control) sows according to ovulation time. The number of sows is shown in brackets. ***, ** and * indicate differences between GnRH and Control for *p* < 0.0001, *p* < 0.01 *p* < 0.05, respectively.

GnRH agonist treatment clearly modified WOI (*p* < 0.001). More specifically, it modified the number of sows that ovulated in the WOIs of 120, 132, 144, 156 and 168 h, causing more sows to ovulate at 120 and 132 h and fewer to ovulate at 144, 156 and 168 h, compared to the controls (Figure 2). Treatment did not affect the fertility results (farrowing rate and litter size) of the inseminated sows, nor of the total number of sows or those that ovulated within the expected time window (Table 2).

### 3.2. Influence of Follicular Size on the Response to GnRH Agonist Treatment

**Figure 3:**
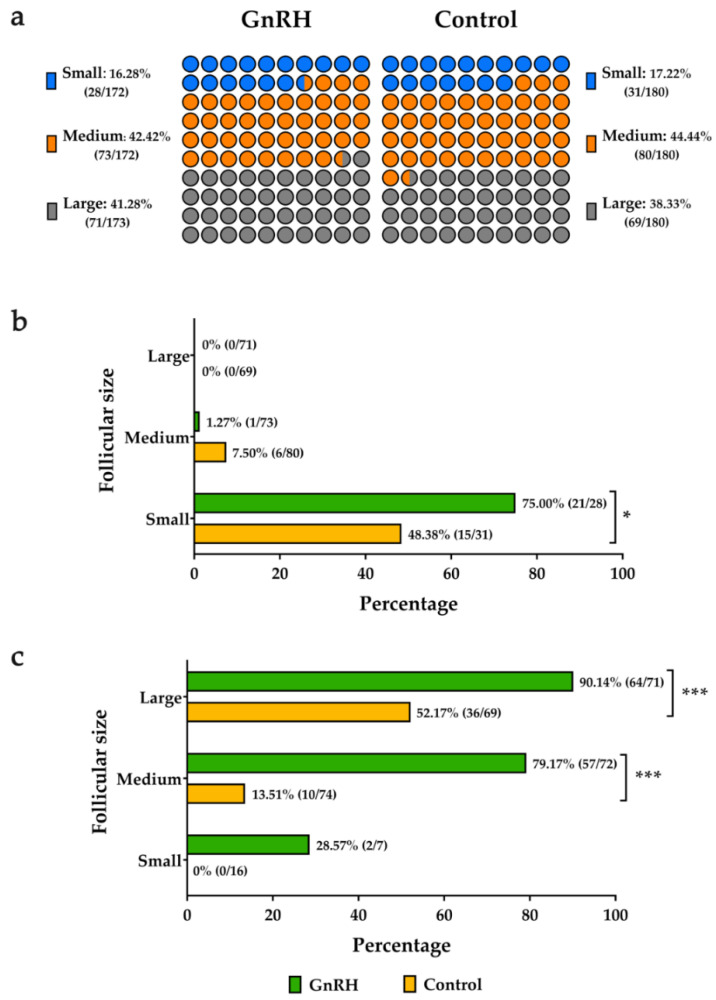
Response to GnRH agonist buserelin was influenced by ovarian follicular size. (**a**) Distribution of GnRH-treated and control sows according to the ovarian follicular size at treatment time. (**b**) Sows in anestrus according to the follicular size at treatment time. (**c**) Sows ovulating within the expected time window according to follicular size at treatment time. Small indicates follicles <0.5 cm, medium between 0.5 and 0.64 cm and large between 0.65 and 1.09 cm. The number of sows is shown in brackets. *** and * indicate *p* < 0.001 and *p* < 0.05 differences, respectively, between GnRH-treated and control sows.

GnRH agonist-treated and control sows were similarly distributed among the three considered clusters of follicular size (Figure 3a). Most sows showed medium and large follicles although a representative number of them showed small follicles. Sows showing small follicles were mainly of one or two parities (57.14% (16/28) in GnRH agonist-treated and 54.84% (17/31) in control sows). Treated and control sows in anestrus were not equally distributed among the three clusters. Among those showing small follicles, the proportion of treated sows in anestrus was higher than within control sows (75.00% (21/28) vs. 48.38% (15/31), respectively; *p* < 0.05) (Figure 3b). The proportion of sows that ovulated within the expected time window (WOI: 120–132 h) increased as follicular size increased, both in the treated and control groups (Figure 3c). The number of sows ovulating in the expected time window was greater among GnRH agonist-treated sows than controls, when considering sows showing medium and large follicles (*p* < 0.001), but not among those showing small follicles, showing figures similar for treated and untreated sows (Figure 3c). Regarding the fertility outcomes of inseminated sows, both farrowing rate and litter size were lower in sows with small follicles than in those with medium or large follicles. This pattern was the same for GnRH agonist-treated and control sows, without differences between groups (Table 2).

### 3.3. Influence of the Period of Weaning on the Response of Sows to GnRH Agonist Treatment

**Figure 4:**
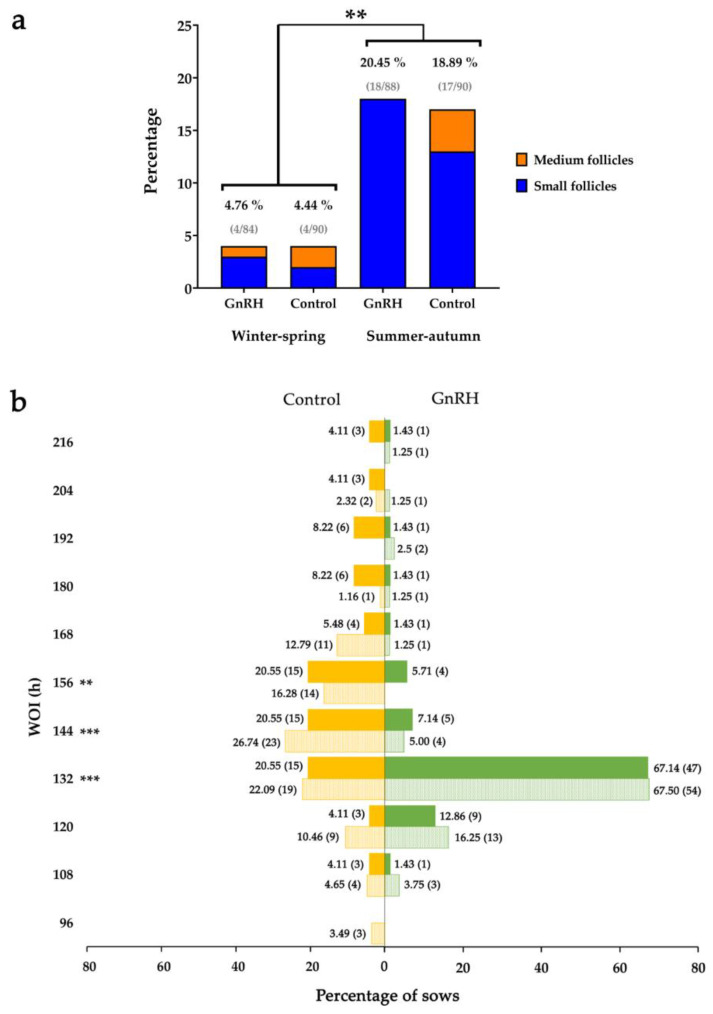
Influence of weaning season on the response to GnRH agonist buserelin. (**a**) GnRH-treated and control sows in anestrus in winter–spring (WS) and summer–autumn (SA) periods. The blue color within the bars indicates the proportion of sows with small follicles at GnRH treatment time (86 h after weaning). ** indicates *p* < 0.01 differences between periods, irrespective of GnRH treatment. (**b**) Distribution of GnRH agonist-treated (green bars) and control (yellow bars) weaned sows according to the weaning-to-ovulation interval in SA (solid bars) and WS (patterned bars). The number of sows is shown in brackets. *** and ** indicate *p* < 0.001 and *p* < 0.01 differences, respectively, between GnRH-treated and control sows, irrespective of the period of the year.

Sows were weaned at two different periods of the year, named SA (July to October) and WS (February to May). The incidence of anestrus was higher (*p* < 0.01) in SA than in WS without differences between GnRH agonist-treated and control sows (Figure 4a). Likewise, the percentage of sows with small ovarian follicles at treatment time was higher (*p* < 0.01) in SA than in WS in both GnRH agonist-treated (22.73% (20/88) vs. 9.52% (8/84) in SA and WS, respectively) and control sows (26.67% (24/90) vs. 7.77% 7/90] in SA and WS, respectively). Many of the weaned sows in anestrus showed small follicles at the time of treatment. Specifically, 95.45% (21/22) of the GnRH agonist-treated and 71.43% (15/21) of the control sows (Figure 4a). The percentage of sows that had ovulated was higher in WS than in SA (*p* < 0.01), without differences between GnRH agonist treatment and controls. Specifically, 95.24% (80/84) and 79.54% (70/88) GnRH agonist-treated sows and 95.56% (86/90) and 81.11% (73/90) control sows ovulated in WS and SA, respectively. The period of weaning did not modify the distribution of sows among various WOIs (Figure 4b). More GnRH agonist-treated sows ovulated at 120–132 h after weaning compared to control sows in both periods of the year (*p* < 0.001), without differences between WS and SA. Regarding fertility outcomes of inseminated sows, both farrowing rates and litter sizes were lower in SA than in WS both in the treated and the control sows, without differences between treated and controls (Table 2).

## 4. Discussion

The percentage of weaned sows that ovulated at the expected time window after administration of buserelin was above 70%. The figures are similar to the percentages achieved in previous studies using buserelin and lecirelin, another GnRH agonist, administered parenterally at a fixed time after weaning [17,19] but higher than in those achieved in studies administering vaginal triptorelin at a fixed time after weaning [20,21]. Taking all these results together, including those of the present study, satisfactory ovulation synchronization rates have been achieved, but are not high enough for an efficient application of a single fixed-time AI. Dillard and Flowers [21], in an experiment with vaginal triptorelin, considered that the large number of weaned sows that did not show estrus in the eight days after weaning impaired the success of pharmacologically synchronizing ovulation. Our results would support this claim, since a substantial percentage of the weaned sows that did not adequately respond to the GnRH agonist treatment remained in anestrus during the eight days after weaning. Furthermore, our results showed that weaned sows that did not show estrus did not ovulate either. Consequently, the percentage of sows that neither show estrus nor ovulate clearly jeopardizes the expected impact of GnRH agonists to adequately synchronize ovulation in weaned sows, pursuing high fertility outcomes after a single fixed-time AI. This reality would raise the question about the role played by GnRH agonist treatment in weaned sows with a tendency to show neither estrous nor ovulation. Weaned sows in anestrus show reduced concentrations of LH [29]. Since the administration of the agonist of GnRH induces an LH surge, it would be reasonably expected that the GnRH agonist treatment would reduce the incidence of weaned sows in anestrus, which was not the case in the present study. The proportion of weaned sows in anestrus was similar between those treated with the GnRH agonist and those remained untreated. Consequently, treatment with GnRH agonists, at least with parenteral buserelin, would not be able to modify the incidence of post-weaning anestrus.

The lack of response of sows not showing post-weaning estrus to GnRH agonist treatment also raises the question whether weaned sows not showing estrus should be treated and further inseminated. Accordingly, administering GnRH agonists only to sows showing signs of estrus instead to all sows at a fixed time after weaning is claimed to be an alternative approach to improve fertility results [30]. Indeed, excellent fertility results have been reported following this approach [8,21]. However, estrus detection does not fit into the concept of a management-efficient single fixed-time AI because it detracts the important benefits planned [30]. Indeed, the administration of GnRH agonists at a fixed time after weaning to synchronize ovulation seeks precisely to eliminate the need of estrus detection, one of the most laborious, exigent and time-consuming farm activities. At this point, it is also interesting to know that administering GnRH agonists at the start of estrus could also worsen the synchronization of ovulation since some sows could experience the endogenous LH surge before it would be induced by the exogenous GnRH agonist and consequently ovulate before the expected time [20]. In this controversy, it is important to point out that the administration of an GnRH agonist is recommended between 83 and 89 h after weaning, time when many weaned sows have already started estrus [10,17].

Interestingly, most sows showing post-weaning anovulatory anestrus had one or two parities. In this regard, Driancourt et al. [17] showed that weaned primiparous sows are less responsive than weaned multiparous sows to the GnRH agonist buserelin. It is also interesting to note that a substantial number of weaned sows with few parities had small ovarian follicles at weaning [23,31]. Sows had shown clear individual differences in average follicular size at weaning [23] and even also by the third day after weaning [32]. In agreement with these previous findings, our results showed that a relevant number of sows had small follicles at GnRH agonist treatment time. In addition, to confirm the already known good responsiveness of weaned sows with large follicles to GnRH agonist treatment [20], our study further demonstrated that the majority of the weaned sows with medium follicle sizes at treatment time also responded well to GnRH agonist treatment as many of them ovulated within the expected time window. However, the most interesting and novel finding of our study was that many of the treated weaned sows with small ovarian follicles did not respond to the GnRH agonist. This finding evidenced that the incidence of sows with small ovarian follicles at treatment time would be a major cause impairing the effectiveness of the GnRH agonist to properly synchronize ovulation in weaned sows. The poor responsiveness by sows with small follicles to the GnRH agonist treatment could be caused by the lack of LH receptors in small follicles, since granulosa cells acquire LH receptors when the follicles reach 0.5–0.6 cm in diameter [33]. The finding that, when treated with GnRH agonist, sows with small follicles showed a higher incidence of anestrus without ovulation than corresponding controls was also interesting. Martinat-Botté et al. [16] also observed that some weaned sows treated with the GnRH agonist buserelin did not show estrus in contrast to the controls where all sows showed estrus. This would indicate that an LH surge at an inappropriate ovarian follicular development time, as would be induced by the GnRH agonist in sows with small follicles, can stop both the full maturation of the follicles and the subsequent ovulation. This clearly shows that GnRH agonist treatments alone will not be able to synchronize ovulation in all treated weaned sows. The presence of weaned sows with small follicles that do not respond adequately to GnRH prevents this. The administration of hormones able of promoting follicular growth before the administration of GnRH agonist, hormones with similar activity to follicle stimulating hormone (FSH), such as equine chorionic gonadotropin (eCG) [34], could be an option to reduce the number of sows with small follicles at GnRH agonist treatment time. In this way, the complete growth of the small follicles would be facilitated, making them responsive to the LH surge induced by the GnRH agonist treatment. The only problem is to properly identify these sows with minimal effort.

In addition to the relevance of ovarian follicular size, the study also explored the putative relevance of season of weaning on the ability of GnRH agonist buserelin to synchronize ovulation in weaned sows. The unfavorable environmental conditions of summer and early autumn in temperate regions, characterized by high air temperatures and large daylength, negatively affects the synthesis and secretion of GnRH and, consequently, that of LH, compromising the full maturation of ovarian follicles and the subsequent ovulation [35]. Thereby, the reproductive performance of sows weaned during SA is worse than those weaned during WS [24,25]. It could be hypothesized that the administration of a GnRH agonist, such as buserelin, could reverse this negative situation and thereby improve the reproductive performance of sows weaned during the summer and early autumn. Unfortunately, our results showed that this did not happen. The percentage of sows that responded to treatment with GnRH agonists was lower in SA than in WS, similarly to what happened in control sows. The presence of a larger number of sows with small follicles at treatment time during SA and the aforementioned poor responsiveness of these sows to GnRH agonist treatment would be the main cause. The positive finding would be that the majority of sows weaned in SA that had ovulated did so within the expected time window, in a similar proportion to those that were weaned during WS. However, this effective synchronization rate did not lead to improvements in fertility outcomes since the farrowing rate and litter sizes of GnRH agonist-treated sows weaned in SA were lower than those weaned in WS and they did not differ than those achieved by control sows.

Looking at fertility outcomes, the purpose of our study was not evaluating the suitability of GnRH agonist treatment for efficient use of a single fixed-time insemination. The GnRH agonist-treated and control sows were inseminated following the same schedule that included two or three intrauterine inseminations during estrus. This approach allowed us to evaluate whether GnRH agonist treatment affected fertility outcomes. Regarding the complete set of GnRH agonist-treated sows, treatment did not affect fertility outcomes of the AI sows, which was expected, considering previous results [8]. GnRH agonist treatments advance ovulation in a number of sows that would physiologically ovulate later. So, it could be expected that the oocytes ovulated in some of these sows were not functionally ready for successful fertilization and/or embryo development. It seems that the oocytes of ovulated GnRH-treated sows were functional, since the fertility results of the GnRH agonist-treated and untreated sows that ovulated in the expected time window were similar, but further studies are warranted to unveil the reasons.

## 5. Conclusions

The study confirmed the effectiveness of the GnRH agonist buserelin to synchronize ovulation within a short time window, in many weaned sows. However, a relevant number of sows were unresponsive to treatment, either because they ovulated later or even did not ovulate. Many of these unresponsive weaned sows had small ovarian follicles at the time of treatment. Therefore, the efficacy of the GnRH agonist buserelin for the successful synchronization of ovulation in weaned sows is still limited by the number of weaned sows that have small follicles at the time of treatment, a number that was greater among sows weaned in summer–autumn than among the sows weaned in winter–spring.

## Figures and Tables

**Table 1:** Characteristics of the weaned sows included in the experiment.

Characteristics	Weaned Sows		Probability
	GnRH	Control	
Body condition ^1^	3.04 ± 0.02	2.99 ± 0.02	Ns ^2^
Lactation period (d)	22.88 ± 0.12	22.87 ± 0.13	ns
Parities			
1	29 (16.86%)	33 (18.33%)	ns
2	44 (25.58%)	35 (19.44%)	
3	23 (13.37%)	28 (15.56%)	
4	30 (17.44%)	35 (19.44%)	
5	21 (12.21%)	28 (15.56%)	
6	25 (14.53%)	21 (11.67%)	
Ovary follicles ^3^			
Diameter (cm)	0.37 ± 0.01	0.37 ± 0.01	ns
Number ^4^	18.15 ± 0.32	18.23 ± 0.31	ns

^1^ Measured over a score range of 1 to 5. ^2^ NS indicates no differences between GnRH agonist-treated and control sows. ^3^ Transrectal scan performed on the day of weaning. ^4^ Number of follicles counted in each ovary.

**Table 2:** Fertility outcomes (as farrowing rate and litter size) as affected by variables between GnRH agonist-treated and untreated control weaned sows.

Variable	Farrowing Rate (%)		Litter Size	
	GnRH	Control	GnRH	Control
All sows	128/150 (85.33%)	139/159 (87.42%)	13.15 ± 0.28	13.15 ± 0.28
Sows ovulating in time window *	105/123 (85.37%)	40/46 (86.96%)	13.25 ± 0.29	13.98 ± 0.36
Ovarian follicular diameter				
Small (<0.5 cm)	6/28 ^a^ (21.43%)	13/31 ^a^ (41.93%)	11.17 ± 1.14 ^x^	12.00 ± 0.89 ^x^
Medium (0.5–0.64 cm)	62/73 ^b^ (84.93%)	65/80 ^b^ (81.25%)	12.68 ± 0.41 ^z^	12.91 ± 0.33 ^z^
Large (>0.64 cm)	60/71 ^b^ (84.51%)	61/69 ^b^ (88.41%)	13.70 ± 0.34 ^z^	13.75 ± 0.33 ^z^
Period of weaning				
Winter–spring	71/128 ^m^ (84.52%)	77/139 ^m^ (85.56%)	13.47 ± 0.30 ^x^	13.62 ± 0.29 ^x^
Summer–autumn	57/128 ^n^ (64.77%)	62/128 ^n^ (68.89%)	12.63 ± 0.46 ^z^	12.66 ± 0.37 ^z^

* Sows ovulation in the expected time window between 120 and 132 h after weaning. ^a,b^ *p* < 0.001; ^m,n^ *p* < 0.01; ^x,z^ *p* < 0.05.
